# Paired Synchronous Rhythmic Finger Tapping without an External Timing Cue Shows Greater Speed Increases Relative to Those for Solo Tapping

**DOI:** 10.1038/srep43987

**Published:** 2017-03-09

**Authors:** Masahiro Okano, Masahiro Shinya, Kazutoshi Kudo

**Affiliations:** 1 Department of Life Sciences, Graduate School of Arts and Sciences, The University of Tokyo, Tokyo, Japan; 2 Graduate School of Interdisciplinary Information Studies, The University of Tokyo, Tokyo, Japan

## Abstract

In solo synchronization-continuation (SC) tasks, intertap intervals (ITI) are known to drift from the initial tempo. It has been demonstrated that people in paired and group contexts modulate their action timing unconsciously in various situations such as choice reaction tasks, rhythmic body sway, and hand clapping in concerts, which suggests the possibility that ITI drift is also affected by paired context. We conducted solo and paired SC tapping experiments with three tempos (75, 120, and 200 bpm) and examined whether tempo-keeping performance changed according to tempo and/or the number of players. Results indicated that those tapping in the paired conditions were faster, relative to those observed in the solo conditions, for all tempos. For the faster participants, the degree of ITI drift in the solo conditions was strongly correlated with that in the paired conditions. Regression analyses suggested that both faster and slower participants adapted their tap timing to that of their partners. A possible explanation for these results is that the participants reset the phase of their internal clocks according to the faster beat between their own tap and the partners’ tap. Our results indicated that paired context could bias the direction of ITI drift toward decreasing.

One of the essential characteristics of humans is coordination with others. Many studies have reported that people coordinate their actions both intentionally and unintentionally^1^,^2^,^3^. Unintentional action coordination has been referred to as interpersonal or social coordination. Interestingly, there is increasing evidence that people modulate or mimic one another’s behaviour in group environments. For example, two people in rocking chairs unintentionally synchronize each sway^4^, people in conversation mimic one another’s gestures and facial expressions^5^, and audiences in concert halls tend to clap in unison^6^. Thus, people often modulate their behaviour unconsciously when they are with other people. In this study, we sought to determine how an environment involving coordination between partners modulated performance in the production of regular time intervals.

The synchronization-continuation (SC) paradigm is often used to explore the ability to produce regular time intervals. In SC tapping tasks, participants are initially required to tap in synchrony with metronome beats, and then continue to tap at the same pace after the metronome has been turned off. Intertap intervals (ITI) in solo SC tapping tasks have been found to drift gradually away from the metronome intervals^7^ (reference ITI). For example, Madison instructed participants to perform solo SC tapping tasks across five tempo conditions with reference ITI ranging from 400 to 2,200 ms. He reported that the proportion of trials in which ITI gradually decreased was larger in the 400 ms condition than in other conditions, in which the amount of positive and negative drift were comparable^8^. Additionally, Collyer *et al*. reported that ITI from 250 to 413 ms tended to decrease, while those from 513 to 748 ms tended to increase^9^. Thus, the appearance of ITI drift in SC tapping tasks varies according to the reference ITI. In most cases, ITI time series show long-period fluctuations in addition to drift^10^,^11^,^12^,^13^,^14^,^15^. For example, Torre *et al*. conducted an SC tapping experiment with a reference ITI of 500 ms; the results provided examples of undulating ITI time series with periods of approximately 50–100 s^15^.

The number of studies examining a paired version of the SC tapping task has increased recently. These studies focused mainly on the local timing correction process. For example, Konvalinka *et al*. conducted paired SC tapping experiments involving uncoupled (neither of participants in a pair could hear their partner’s beats), unidirectional coupling (only one of the participants in a pair could hear their partner’s beats), and bidirectional coupling (both of the participants in a pair could hear their partners’ beats) conditions^16^. They found significant negative lag-0 and positive lag ± 1 cross-correlations between ITI of partners in each pair in the bidirectional coupling condition. They described the partners as ‘hyper-followers’, that is, both partners followed the other’s prior tap. In addition, the results showed a negative trend in the ITI time series for typical examples in the bidirectional coupling condition. However, the authors did not discuss ITI drift because the focus of the study was the relationship between coupling properties and ITI variability. Further, the number of taps in a single trial was limited to 32 (no more than 20 s), which was too short to allow assessment of ITI drift over longer timescales, such as those of full musical pieces that continue for several minutes. In other studies involving paired SC tapping or similar tasks, task durations were limited to several tens of seconds^17^,^18^,^19^,^20^,^21^,^22^,^23^. These durations are insufficient to judge whether ITI drift in paired SC tasks is similar to that in solo SC tasks, in which ITI time series display long periods of fluctuation (for as long as 50–100 s^15^). The only study we identified with sufficient trial duration was a paired tapping experiment conducted by Hennig^24^, in which participants continued to tap for almost 8 min. All the data in his paper shows negative trend in ITI time series. However, Hennig instructed participants to synchronize their tapping at a comfortable tempo. Therefore, the task demands differed from those of SC tapping tasks, in which players were required to maintain the reference ITI.

Therefore, previous studies show that ITI tends to decrease during paired SC tapping tasks; however, it is unclear how ITI drift behaves when participants intend both to maintain the reference tempo and synchronize their tapping with that of their partners for several minutes. In this study, we conducted solo and paired SC tapping experiments, to determine whether the behaviour of the ITI drift (1) changes according to tempo (75, 120, and 200 beats per minute (bpm); 800, 500, and 300 ms in ITI, respectively), and (2) differs between solo and paired conditions. In the experiment, participants were asked to tap with keeping the initial tempo as much as they could. The duration of each trial was 200 s, during which participants tapped in synchrony with the metronome beats for the first 10 s and continued to tap without the metronome beats for the remaining 190 s, in both the solo and paired conditions. In the paired conditions, participants were asked to synchronize their tapping with that of their partners, in addition to maintaining the reference tempo. The participants could hear their partners’ tapping but could not see it. The results suggested that tapping paces gradually accelerated in paired conditions, while tapping paces fluctuated around the initial paces in solo conditions (Fig. 1). The question is which processes generated the gradual acceleration in paired conditions. One possible answer is that unidirectional leadership drove the acceleration of tapping pace: that is, the participants who tended to tap at a faster pace (faster participants) than their partners (slower participants) in solo conditions acted as leaders of each pair and the slower participants as followers. If that were the case, taps of faster participants would tend to precede taps of the partners and the taps of slower participants would follow the faster partner’s taps with a lag unidirectionally. However, tap timing asynchrony (difference between tap timing of partners) fluctuated around zero (Figures S1–7 in supplementary materials) and ITI time series in paired conditions displayed antiphase, i.e. a ‘hyper-followers’ pattern (Figure S8). Therefore unidirectional leadership did not appear to explain our data. The alternative explanation is that bidirectional timing modulation between partners caused the acceleration in paired conditions. Sensorimotor synchronization literatures suggest that participants modulate their tap timing to keep synchronization with pacing sequences or partners (phase correction)^7^,^25^. Phase correction response occurs to a greater extent toward preceding distractor tones that sound immediately before target tones than toward delayed distractors that sound immediately after targets^26^. In addition, participants generally tap tens of milliseconds before target tones in sensorimotor synchronization tasks (negative mean asynchrony: NMA)^7^. A combination of phase correction asymmetry and NMA appears to drive tapping pace acceleration for ‘hyper-follower’ participants. If that is the case, the degree of their tap timing modulation based on timing error would be equivalent regardless of their pace in solo conditions. To test this hypothesis, we performed single and multiple regression analyses to determine whether, and to what extent, the interaction between partners affected the ITI change rate.

## Results

### Comparison of performance between solo and paired conditions

**Figure 1:**
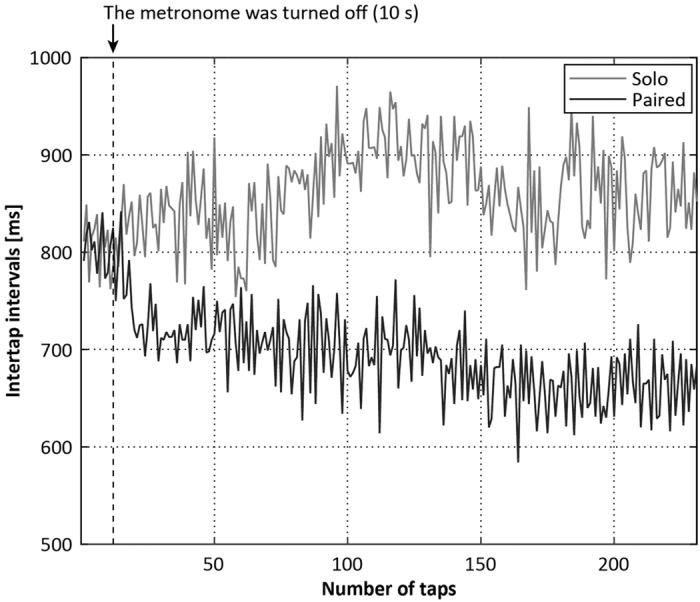
Typical ITI time series for the solo and paired conditions (75 bpm). In the solo conditions, ITI fluctuated around the reference ITI (800 ms above). In contrast, ITI decreased over time in paired conditions.

**Figure 2:**
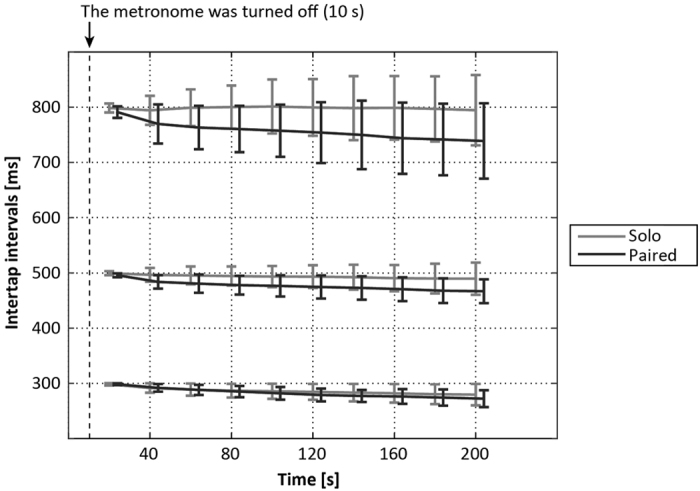
Mean ITI transition every 20 seconds across all pairs (mean ± SD). In 75 bpm (800 ms) conditions, solo ITI remained close to the reference ITI on average, while paired ITI decreased monotonically. In the 120 and 200 bpm (500 and 300 ms, respectively) conditions, both solo and paired ITI decreased gradually, and paired ITI decreased further.

**Figure 3:**
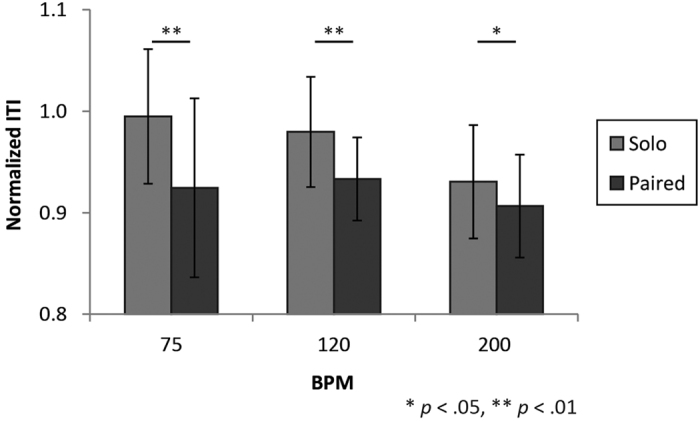
Comparison of mean normalized ITI for the final 31 taps (mean ± SD). Normalized ITI were obtained by dividing measured ITI by the corresponding reference ITI (800, 500, and 300 ms for 75, 120, and 200 bpm, respectively). ITI in the paired conditions were significantly shorter relative to those observed in the solo conditions, regardless of tempo.

Although 26 people (13 pairs) participated in the experiments, one participant was unable to synchronize with the partner. We excluded the data for this pair of participants; therefore, the following results are based on data from the remaining 12 pairs. Figure 1 provides typical examples of ITI time series in the solo and paired conditions. Figure 2 shows a summary of the mean ITI transition every 20 s. Mean ITI decreased gradually as time elapsed in the paired conditions, and oscillated around the reference ITI in the solo conditions. This tendency was consistent across trials (Figure S9). We conducted a two-way repeated measures ANOVA to compare means for the final 30 ITI in the solo and paired trials, with tempo and number of participants (i.e., solo and paired) as factors, to determine whether the degree to which ITI decreased differed between conditions (Fig. 3). The ANOVA revealed significant main effects of number of participants, *F*(1, 23) = 23.65, *p* < 0.001, and tempo, *F*(2, 46) = 10.02, *p* < 0.001, and a significant interaction between these factors, *F*(2, 46) = 3.88, *p* = 0.028. *Post-hoc* tests revealed significant differences between solo and paired conditions for all tempo conditions: 75 bpm: *t*(23) = 3.86, *p* = 0.001; 120 bpm: *t*(23) = 4.06, *p* < 0.001; 200 bpm: *t*(23) = 2.46, *p* = 0.022. In solo conditions, the mean ITI for the final 30 taps differed significantly between 75 and 200 bpm, *t*(23) = 4.47, *p* < 0.001, and between 120 and 200 bpm, *t*(23) = 4.53, *p* < 0.001. In the paired conditions, a significant difference was observed only between 120 and 200 bpm, *t*(23) = 2.68, *p* = 0.014.

### Relationship between performances in the solo and paired conditions

**Figure 4:**
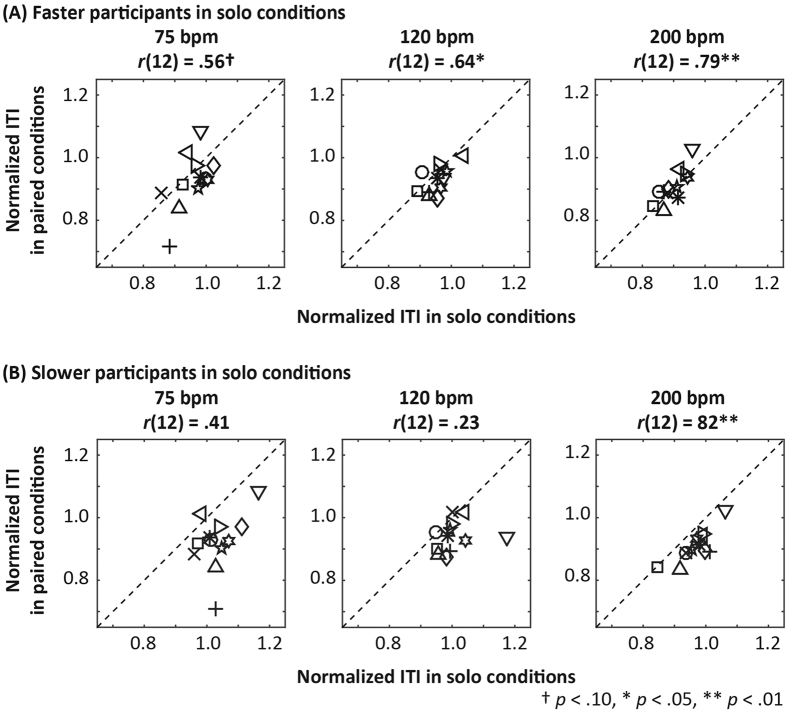
Correlations between normalized ITI for the final 31 taps in the solo and paired conditions. Across all panels, different markers represent participants from different pairs, and identical markers represent participants from the same pair. In this figure, the participants in each pair were classified into two groups: those who tapped faster and those who tapped slower in the solo conditions. (**A**) The faster participants’ ITI in the solo conditions were marginally or significantly correlated with the ITI in the paired conditions. They are plotted in the vicinity of the identification line (dotted line), indicating that the faster participants tapped at almost the same pace in both the solo and paired conditions. (**B**) In contrast, the slower participants’ ITI are plotted below the identification line, indicating that their tapping pace in the paired conditions was faster relative to that observed in the solo conditions.

The results described above suggest that ITI tended to decrease in the paired conditions. This raised a question as to whether there would be a correlation between the degrees of ITI drift in the solo and paired conditions. Within each pair, participants could be divided into those who tended to tap faster (the faster participants) and those who tended to tap slower (the slower participants) in the solo conditions. Therefore, we examined correlations between means for the final 30 ITI in the solo and paired conditions, dividing the participants in each pair into the faster and the slower groups, based on means for the final 30 ITI in the solo conditions (Fig. 4). In Fig. 4, faster participants’ means for the final 30 ITI were plotted in the vicinity of the identification line. In contrast, most of the slower participants’ mean ITI were plotted below the identification line. This indicated that faster participants tapped at almost the same pace in both the solo and paired conditions, while slower participants’ pace in the paired conditions was faster relative to that in the solo conditions. For faster participants, significant correlations between the means for the solo and paired conditions were observed for 120 bpm, *r* (12) = 0.64, *p* = 0.03, and 200 bpm, *r* (12) = 0.79, *p* < 0.01, while a marginally significant correlation was observed for 75 bpm, *r* (12) = 0.56, *p* = 0.06. In slower participants, the correlation between the means for the solo and paired conditions was significant for 200 bpm, *r* (12) = 0.82, *p* < 0.01, while those for 75 and 120 bpm were nonsignificant, *r* (12) = 0.41, *p* = 0.19 and *r* (12) = 0.23, *p* = 0.48, respectively.

### Leadership or interactive modulation: which caused ITI decrease?

**Figure 5:**
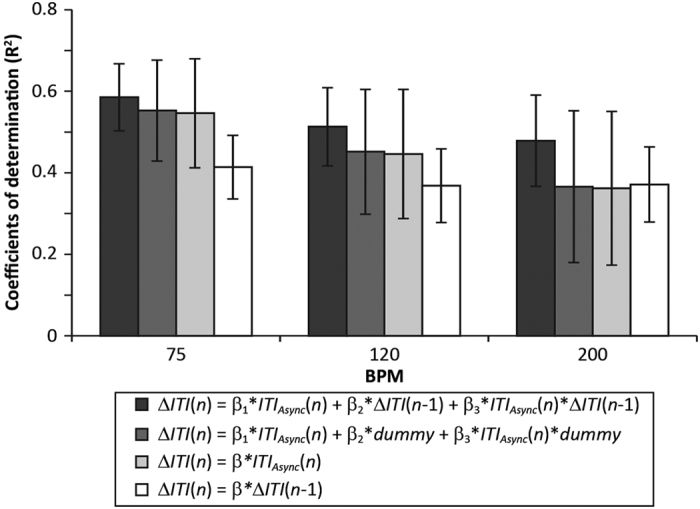
Coefficients of determination (R^2^) for the paired conditions, obtained via regression analyses. The bars represent R^2^ values obtained from corresponding models (mean ± SD); “dummy” denotes dummy variables composed via the randomly permutated Δ*ITI (n* − 1). As tempo decreased, the contribution that the Δ*ITI (n* − 1) made to R^2^ decreased; in other words, the relative importance of the *ITI*_*Async*_ (*n*) increased. This would be related to the margin within which participants adapted their tap timing to that of their partners. Note that R^2^ always increased, even when dummy variables were added, in the multiple regression model comparing to the single regression model, which make *post-hoc* tests find “significant” difference between the light gray and white bars; however, this difference was extremely small. The differences in effect sizes (Cohen’s *d*) between black and light gray bars should be noted; these differences were 0.31, 0.49, and 0.70 for 75, 120 and 200 bpm, respectively. Those observed between the light gray and white bars were smaller at 0.05, 0.04 and 0.02 for 75, 120 and 200 bpm, respectively.

**Figure 6:**
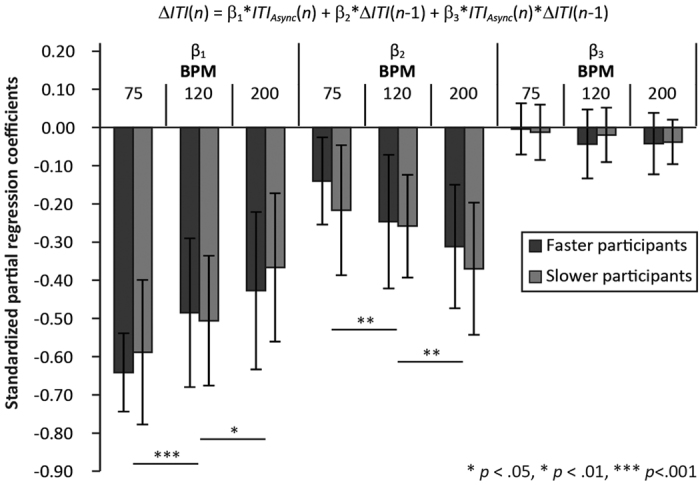
Standardized partial regression coefficients for each tempo in the paired conditions. The multiple regression model is as follows: ∆*ITI (n*) = β_1_ * *ITI*_*Async*_ + β_2_ * ∆*ITI (n* *−* 1) + β_3_ * *ITI*_*Async*_ * ∆*ITI (n* *−* 1). The greater the tapping rate increase, the greater the relative importance of the decrease in β_1_, which would correspond to the margin within which participants adapted their timing to that of their partners. The β values did not differ significantly between faster and slower participants.

Correlation analysis suggested that the degrees to which ITI decreased in the paired conditions were comparable to those observed for the faster participants. The next question was which caused the decrease in the ITI in paired conditions, leadership of faster participants or interactive timing modulation between partners. Tap timing asynchrony between partners fluctuated around zero (Figures S1–7) and ITI time series in paired conditions displayed “hyper-follower” pattern (Figure S8). These support the hypothesis that interactive timing modulation brought the ITI decrease. To confirm this, we performed single and multiple regression analyses to determine whether the ITI change rate (*∆ITI*) for the entire duration of each trial was affected by the interaction between participants in the pair. We set the *∆ITI (n*), which was derived by subtracting the *n*th ITI from the (*n* + 1)th ITI for one participant (i.e., intrapersonal modulation), as the dependent variable and the *∆ITI (n* − 1) and the *ITI*_*Async*_ (*n*), which was derived by subtracting the *n*th ITI for the partner from the *n*th ITI for the participant (i.e., interpersonal modulation), as explanatory variables. Note that the *ITI*_Async_ (*n*) occurs immediately prior to the *∆ITI (n*) because of its definition (see equations (1) to (3), in the Methods section and Figure S10 for details). Single regression analyses revealed that the mean determination coefficients (R^2^) for the *∆ITI (n* *−* 1) were 0.41, 0.37, and 0.37 for 75, 120, and 200 bpm, respectively. Meanwhile, the mean R^2^ values for the *ITI*_*Async*_ (*n*) were 0.55, 0.45, and 0.36 for 75, 120, and 200 bpm, respectively. To determine which explanatory variable exerted the stronger effect on the *∆ITI (n*), we conducted a two-way repeated measures ANOVA to compare R^2^ values between the *∆ITI (n* *−* 1) and *ITI*_*Async*_ (*n*), with tempo and type of explanatory variable as factors. The ANOVA revealed significant main effects of tempo, *F*(2, 94) = 26.34, *p* < 0.001, and type of explanatory variable, *F*(1, 47) = 13.90, *p* = 0.001, and a significant interaction between the two factors, *F*(1.72, 80.90) = 21.19, *p* < 0.001. *Post-hoc* tests revealed that R^2^ values for the *∆ITI (n* *−* 1) and *ITI*_*Async*_ (*n*) differed significantly at 75 bpm, *t*(47) = 7.21, *p* < 0.001, and 120 bpm, *t*(47) = 3.40, *p* = 0.001, whereas not at 200 bpm, *t*(47) = 0.21, *p* = 0.65. A significant main effect of tempo was observed for both the *∆ITI (n* *−* 1) and *ITI*_*Async*_ (*n*), *F*(2, 94) = 6.22, *p* = 0.003 and *F*(1.78, 83.51) = 30.09, *p* < 0.001, respectively. R^2^ did not differ significantly between faster and slower participants in each pair (see supplementary analysis and Figure S11 in supplementary materials for detail). Multiple regression analysis was then performed using the forced entry method to determine whether the increase in R^2^ was significant, and compare standardized partial regression coefficients β_1_, β_2_, and β_3_ for the *ITI*_*Async*_ (*n*), *∆ITI (n* − 1), and *ITI*_*Async*_ (*n*) * *∆ITI (n* *−* 1), respectively. The results are summarized in Table 1 and Fig. 5: adjusted R^2^ values were 0.59, 0.51, and 0.48 for 75, 120, and 200 bpm, respectively. In general, R^2^ increases slightly even when adding meaningless variables such as random numbers; therefore, we performed a permutation test using dummy variables composed via the randomly permutated *∆ITI (n* *−* 1), to determine whether the increase in R^2^ was significant (Fig. 5; see the Methods section for details). One-way repeated measures ANOVAs on the adjusted R^2^ were conducted separately for each tempo condition (*a priori* comparison), with regression model type (a): single, (b): multiple with dummy variables, and (c): multiple with the original *∆ITI (n* *−* 1) as the factor. The results revealed significant main effects of regression model type, *F*(1.02, 47.71) = 13.64, *F*(1.01, 47.35) = 24.10, and *F*(1.01, 47.41) = 44.04 for 75, 120, and 200 bpm, respectively (*ps* < 0.001). *Post-hoc* tests revealed significant differences between regression model types for each tempo condition: (a) versus (b): Cohen’s *d* = 0.05, 0.04, and 0.02 for 75, 120, and 200 bpm, respectively (*ps* < 0.01); (a) versus (c): Cohen’s *d* = 0.31, 0.49, and 0.70 for 75, 120, and 200 bpm, respectively (*ps* < 0.001). Regarding βs, as tempo increased, the absolute value of β_1_ decreased and the absolute value of β_2_ increased (Table 1). If the reason for the negative ITI drift was that the faster participant in each pair led the slower participant, the absolute value of β_1_ (interpersonal modulation) for the slower participant would be higher relative to that of the faster participant. To test this hypothesis, we compared β values between the faster and slower participants in each pair. Two-way mixed-design ANOVAs were performed on β_1_, β_2_, and β_3_ separately, with participant type (faster and slower participants) and tempo as factors. The results are shown in Fig. 6: The main effect of participant type was nonsignificant, *F*(1, 46) = 0.71, *F*(1, 46) = 2.29, and *F*(1, 46) = 0.19 for β_1_, β_2_ and β_3_, respectively (*ps* > 0.05). The main effect of tempo was significant for β_1_ and β_2_, *F*(2, 92) = 20.81 and *F*(1.63, 75.1) = 15.41, respectively (*ps* < 0.001), and nonsignificant for β_3_, *F*(2, 92) = 2.66, *p* = 0.08. The interaction between the two factors was nonsignificant for all β values, *F*(2, 92) = 0.88, *F*(2, 92) = 0.66, and *F*(2, 92) = 0.63 for β_1_, β_2_, and β_3_, respectively (*ps* > 0.10).

## Discussion

The purpose of this study was to examine the effects of tempo and number of participants on ITI drift. Our results indicated that the extent to which ITI drifted in a negative direction from the reference ITI for the paired conditions was greater than that for the solo conditions, within a tempo range of 75–200 bpm (the ITI range of 800–300 ms). Previous studies using solo SC tapping suggested that tapping pace increased with ITI of 300 ms^9^, while the results of the current study indicated that, within an ITI range of 300–800 ms, the increase in tapping pace in the paired conditions was greater than that in the solo conditions. In addition, the long-period of oscillation around the reference ITI, which is typically observed in solo SC tapping tasks^15^, was not observed in the paired conditions. Rather, the ITI time series in the paired conditions showed monotonic negative trends during the 200 s in each trial. Significant linear correlations were observed between the faster participants’ means for the final 30 ITI in the solo and paired conditions (Fig. 4). The degree to which ITI decreased in the solo conditions in this group was almost equal to that in the paired conditions. The linear correlation for slower participants was weaker than that for faster participants and was not always significant. These results suggest that the degree to which ITI decreased was dependent mainly on the faster participant in each pair. However, the results from single and multiple regression analyses suggested that both participants in each pair contributed to the acceleration of paired tapping. Interpersonal ITI difference (*ITI*_*Async*_) exerted a significant effect on intrapersonal timing modulation (*∆ITI*), which indicated that participants adjusted their tapping timing to follow that of their partners. The R^2^ value for the *ITI*_*Async*_ (*n*) increased as the tempo decreased, which suggested that participants had a greater margin for which they adapt their timing to that of their partners in slower conditions. This was replicated in the multiple regression analyses: the relative importance of the *ITI*_*Async*_ (*n*) increased as tempo decreased (Figs 5 and 6). In particular, β values did not differ significantly between the faster and slower participants in each pair, regardless of tempo, which indicated that the degrees of inter- and intrapersonal timing modulation did not differ significantly between the two groups. The negative sign of the regression coefficient for the *ITI*_*Async*_ (*n*) indicated that participants prolonged subsequent ITI when their current ITI were shorter than those of their partners, and *vice versa*. Therefore, ITI decrease appeared to have occurred not only because faster participants led slower participants unidirectionally but also via an interpersonal timing adaptation process. Although the number of studies involving paired tapping tasks has increased, none examined differences in ITI drift between solo and paired SC tapping tasks in which each trial lasts for several minutes. Many previous studies examining social interaction and interpersonal coordination demonstrated the modulation of human self-paced movement timing via interpersonal interaction^16^,^27^; however, no studies have demonstrated such a drastic and monotonic drift observed under the instruction to maintain the initial tempo in the current study.

In general, 1/*f* noise is a potential cause of ITI drift in solo SC tasks^7^. The 1/*f* noise has a large low-frequency component and is ubiquitous in biological signals, such as walking^28^ and running strides^29^,^30^, body sway in quiet standing^31^, and heartbeat^32^,^33^, in addition to rhythmic finger-tapping tasks^34^,^35^. However, it cannot explain the drift in the paired SC task in the current study because the drift caused by 1/*f* noise does not occur in a specific direction: most of the time series displayed a simple negative trend in the current study.

What caused the increase in drift in the paired trials? It is possible that the participants in each pair reset the phase of their internal clocks according to their planned tap timing and their partners’ actual tapping. Indeed, perturbation studies reported that participants’ tap timing was strongly attuned to distractor tones, particularly when they preceded target tones^36^,^26^,^37^. This process would explain the significant correlation between ITI drift in the paired conditions and solo conditions observed in the paired conditions in faster participants. Similar to the decrease in ITI, force exertion is known to escalate in paired contexts^38^. One explanation for force escalation is that self-generated forces are perceived as weaker relative to externally generated forces of the same magnitude, because of the predictive process involved in motor control^38^. This process is concerned with enhancing the salience of sensations from external stimuli^39^,^40^,^41^. Assuming that this process occurs, the combination of reduced sensitivity to timekeeping and a tendency to be attracted to the preceding beat is likely to reduce ITI in paired contexts. On the other hand, the participants in the current experiment could be considered coupled oscillators. Sufficiently strongly coupled oscillators often entrain one another; when the coupling is bidirectional, they oscillate at a frequency between the natural frequencies for each oscillator, whereas when the coupling is unidirectional, one oscillator is forced to oscillate at the natural frequency of the other^42^. In the paired conditions in the current study, tapping occurred at almost the same pace as that of the faster participant in each pair in the solo conditions (Fig. 4). In this sense, the faster participant in each pair would have been considered the leader, even though the experimenter did not assign the roles of leader and follower to participants. If a leader-follower relationship existed, it would have emerged spontaneously during the paired task. However, β values for the *ITI*_*Async*_ (*n*) did not differ significantly between the faster and slower participants in each pair (Fig. 6), which suggests that both participants adapted their tap timing to that of the other to almost the same degree; that is, they behaved as equivalent subsystems within the pair. Further research should be conducted to determine the type of interactions that exist.

Is there an ecological explanation for the finding that participants’ tapping in the paired conditions was faster relative to that observed in the solo conditions? Negative mean asynchrony (NMA), which is a tendency to respond a few tens of milliseconds earlier than the metronome beat, is known as a typical behaviour in synchronization tasks involving metronome beats^7^,^25^. NMA is considered to be related to participants’ intention to reduce the variability of synchronization^7^. One explanation for NMA is that participants synchronize sensory feedback from their tapping with the metronome beat, rather than their own tap timing, to facilitate the detection of large asynchronies^43^. On the other hand, NMA is also considered as an anticipatory reaction to the periodic cue. Is it possible to react in an anticipatory manner to sequences with random variability? The results of a study conducted by Washburn *et al*. indicated that this was possible, even with enhanced perceptual delay^44^; although the task used in the study involved circle drawing rather than tapping. If the participants in the current study had regarded their partners’ tapping as a pacing sequence, NMA, the phase resetting mentioned above, and the lack of a credible clock could have resulted in the increase in the tapping pace. Considering the above, an increased tapping pace would be an undesired consequence of overadaptation resulting from automatic anticipation or a strategy for achieving the common goal of synchronization. In this sense, our results could be considered to reflect a combination of cooperation and interdependence within each pair. If so, it would be interesting to examine the relationship between task performance and the social relationships between the participants in pairs. Paired SC tapping would be an interesting paradigm via which to investigate the dynamics of temporal tasks within a group, such as musical ensembles. Indeed, the tempo of ensembles tends to increase as they play, which is known as *rushing*^45^. *Rushing* is thought to occur because of certain personal properties, such as excitement, poor sense of rhythm, or a lack of training, in the players^45^. In contrast, our results suggest that an external factor, the ensemble environment itself, could be the source of *rushing*.

Which neural correlates do underlie performance in the paired SC task? The cerebellum and basal ganglia are considered to make the greatest contribution to the production of the subsecond interval timing used in our study, in both paced and self-paced timing tasks^25^. Significant activity in the prefrontal-parietal-temporal network^46^ and connectivity in the beta band in the mesial-central area^47^ are characteristic of the brain activity observed during solo self-paced, rather than externally paced, tapping. However, few studies have been conducted to improve understanding of characteristic brain activity during paired tapping. A neuroimaging study involving an adaptive virtual partner revealed that significant activation in medial areas, including the posterior cingulate, precuneus, hippocampus, ventromedial prefrontal cortex, and supplementary motor area, was observed when participants felt that they were in synchrony with their virtual partners, and this was not observed for solo tapping^19^,^25^. Activation in these cortical midline regions is considered to be associated with feelings of successful cooperation and the comfortable socioemotional experience^19^ that results from this success. The study also revealed that lateral-frontal regions, including the anterior insula, inferior and superior frontal gyri, ventrolateral prefrontal cortex, and inferior parietal lobe, showed significant activation when the participants felt out of sync^19^. Activation in these regions is considered to be associated with task difficulty and cognitive control^19^. The results concerning ITI drift in the current study suggest a failure to match movement to the reference ITI. It is possible that partners’ tapping served as a cue, which could have reduced participants’ cognitive load and activation in the right lateral-frontal regions. In addition, previous studies demonstrated that participants who led their partners, or felt that they had done so, showed right lateral-frontal activation^20^,^48^. However, our task took a relatively long time to complete; therefore, it is possible that the leader-follower relationship between participants in each pair changed occasionally. If so, what occurred in the nervous system when the relationship changed? The answer to this question could facilitate further understanding of the dynamics of social interaction.

In conclusion, our study revealed that the durations of ITI tended to decrease in pairs performing an SC tapping task. This is the first study to focus on ITI drift in a paired SC tapping task lasting several minutes. Phase resetting would have caused ITI decrease; however, further investigation is required to determine the underlying process. Drift has been regarded as an inherent property of central timekeeper^7^. Ogden and Collier reported that stochastic, rather than deterministic, drift appeared to be an important component of the drift observed in solo SC tasks^49^; therefore, previous studies have often removed the effects of drift by detrending, differentiating, or using an enhanced Wing-Kristofferson model^7^,^50^,^51^, and few mathematical model studies have examined the causes of drift. However, the results of the current study indicated that in some cases, such as those involving a paired context, could cause deterministic and reproducible ITI drift towards decrease. This paradigm would serve as an interesting means via which to gain knowledge regarding mutual timing modulation and social interaction.

## Method

### Participants

26 healthy adults (13 pairs; mean age = 24.2 years, SD = 2.2 years) participated in the experiment. Only two participants reported musical expertise; they were amateur drummers who had played for 5 and 10 years. All participants provided informed consent for their participation in the experimental procedures, which were conducted in accordance with the Declaration of Helsinki and approved by the ethics committee of the Graduate School of Arts and Sciences at the University of Tokyo.

### Apparatus

Two electric drum kits (WAVEDRUM Mini, KORG, Tokyo, Japan) were used to measure participants’ tap timing and produce sound feedback. Participants tapped WAVEDRUM sensors (with a 9.3 cm × 6.5 cm hard plastic board pasted on top for easy tapping) located in front of them, and the corresponding voltage was sampled at 1,000 Hz, using a 16-bit analog-to-digital converter (USB-6218 BNC, National Instruments, Austin, TX, USA). The monitor facing participants showed the time remaining in each trial, using a progress bar programmed via LabVIEW (National Instruments). Participants could hear the metronome beats through a speaker located beneath the monitor, and their drum sounds and those of their partners were played through the speakers. The metronome beats were presented using an application (Metronome Beats 2.3., www.stonekick.com) on the experimenter’s smartphone (ZenPhone5, ASUS) and sampled at 1,000 Hz using the same analog-to-digital converter. Partitions were used to separate the participants in each pair from each other and the experimenter, to prevent visual contact and possible distraction resulting from the experimenter’s gaze.

### Procedure

The participants entered the laboratory in pairs and sat on chairs in front of the experimental materials, and the experimenter explained the procedure. Participants practiced tapping to obtain appropriate intensity with a good signal-to-noise ratio prior to beginning the trials. During the solo trials, one participant tapped in synchrony with the metronome beats, and the experimenter initiated measurement. Ten seconds subsequent to measurement initiation, the metronome was switched off, and the participant continued tapping as closely as possible to the reference ITI. The other participant rested until his or her partner had completed the trial. The reference ITI were counterbalanced and set at 800, 500, and 300 ms (in bpm, 75, 120, and 200 bpm, respectively). These settings were intended to cover slow, medium, and fast musical tempo as well as replicate those used in previous studies; the ITI series in a solo 800 ms condition should remain the same or increase, while those in the solo 500 and 300 ms conditions should decrease^8^,^9^. Comparisons between these three conditions probably contribute to potential interactions between tempo and the number of participants. The duration of each trial was set at 200 s, which was considered sufficient for the long-term ITI fluctuation observed in a previous study^23^. Soon after one participant completed a trial, the other participant began a solo trial, following the same procedure. After both participants had completed all of the trials in the first solo block, the first paired block began. In the paired blocks, participants were required to tap in synchrony with each other and maintain the reference ITI as much as possible, as if they were members of an ensemble. Participants completed the second solo, second paired, and third solo blocks in the same manner, with 24 trials in total. The time required to complete all trials was approximately 100 min.

### Tap Onset Detection

We preprocessed data to optimize tap onset detection, using the following procedure: We obtained the zero-mean voltage time series by subtracting the mean value from the original time series, and performed full-wave rectification. Data were passed through a 100–300 Hz second-order Butterworth band-pass filter. Filtered data were Hilbert transformed, and the instantaneous amplitude was calculated to obtain an envelope for the waveform. The tap onset times in the preprocessed data were defined as the times at which the data value was larger relative to that of the threshold, and the all values of the adjacent 50-ms window were smaller relative to that of the threshold. The threshold was set at a value equivalent to the percentile rank of 95% from the lowest of the preprocessed data. We also implemented an algorithm to ignore the prospects of tap onset that were close (<60% of the reference ITI) to actual onset, to avoid detecting unintended consecutive hits.

### Statistics

We obtained the ITI time series by subtracting the tap onset time from the adjacent tap onset time (Equation 1). ITI were divided using a corresponding reference ITI (normalized ITI), and the normalized ITI were averaged across 30 ITI windows. Before comparing normalized ITI across conditions, we verified whether the normalized ITI differed between trials within each condition (see Supplementary Materials for details). Thereafter, a two-way repeated measures ANOVA, with tempo and number of participants as factors, was performed to compare means for the final 30 ITI in the trials in each condition. *Post-hoc* Bonferroni tests were performed to compare the factors for which a significant effect was observed. All ANOVAs were performed using SPSS version 20.0 for Windows (IBM Japan, Tokyo, Japan).

We also calculated correlation coefficients for the relationships between the means for the final 30 ITI in the solo and paired conditions, classifying the participants in each pair into the faster and slower groups based on the final 30 ITI in the solo trials.

Single and multiple regression analyses were performed to determine whether and the extent to which the interaction between participants in a pair contributed to the modulation of their tap timing, and examine the extent of this relationship. The dependent variable was the *∆ITI (n*) and the explanatory variables were the ∆*ITI (n* − 1) and *ITI*_*Async*_ (*n*). These variables and the ITI were defined using tap timing, as follows:




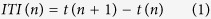







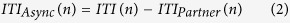







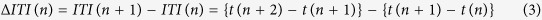




where *ITI (n*), *t (n*), ∆*ITI (n*), *ITI*_*Async*_(*n*), and *ITI*_*Partner*_(*n*) denote the *n*th value of the ITI, tap timing, ∆*ITI*, *ITI*_*Async*_, and *ITI (n*) for the partner, respectively. Note that the moment at which the ∆*ITI (n*) arises is the moment at which the (*n* + 2)th tap occurs (equation (3)); therefore, the index for the *ITI*_*Async*_ immediately before the ∆*ITI (n*) is *n* (Figure S10). The R^2^ value shown in the single regression analysis was higher when using the *ITI*_*Async*_ (*n*); therefore, we sought to determine the extent of the increase in R^2^ in a comparison between multiple and single regression analyses. We performed multiple regression analysis using the *ITI*_*Async*_(*n*) and randomly permutated the ∆*ITI (n* *−* 1) as a dummy variable 1,000 times in each trial (a permutation test), in addition to using the *ITI*_*Async*_(*n*) and the original ∆*ITI (n* *−* 1). The means of the 1,000 adjusted R^2^ values obtained via the permutation test in each trial were compared with those obtained via single regression analysis using the *ITI*_*Async*_(*n*) and multiple regression analysis using the original ∆*ITI (n* *−* 1). A two-way repeated measures ANOVA was performed, with tempo and regression model type as factors, to compare R^2^ values. Prior to the ANOVA, we calculated the positive square root of R^2^ and performed *z*-transformation. In addition, to determine whether the partial regression coefficients differed between the faster and slower participants in each pair in the solo conditions, we performed a two-way mixed ANOVA, with tempo and participant group (the faster and the slower participants in each pair) as factors, for each β value. The level of significance was set at 5% for all statistical tests. A Greenhouse-Geisser correction was used to assess sphericity. All *post-hoc* tests for multiple comparisons were performed using the Bonferroni method.

## Additional Information

**How to cite this article:** Okano, M. *et al*. Paired Synchronous Rhythmic Finger Tapping without an External Timing Cue Shows Greater Speed Increases Relative to Those for Solo Tapping. *Sci. Rep.* **7**, 43987; doi: 10.1038/srep43987 (2017).

**Publisher's note:** Springer Nature remains neutral with regard to jurisdictional claims in published maps and institutional affiliations.

## Supplementary Material

Supplementary Materials

## Figures and Tables

**Table 1:** Standardized partial regression coefficients calculated via multiple regression analysis.

		R^2^	β_1_	β_2_	β_3_
75 bpm	Mean	0.59	−0.61	−0.18	−0.01
	Max	0.80	−0.13	0.02	0.23
	Min	0.37	−0.87	−0.64	−0.25
120 bpm	Mean	0.51	−0.50	−0.25	−0.03
	Max	0.69	0.03	0.11	0.12
	Min	0.23	−0.81	−0.66	−0.32
200 bpm	Mean	0.48	−0.40	−0.34	−0.04
	Max	0.77	0.07	−0.01	0.19
	Min	0.27	−0.80	−0.70	−0.19

Note. The regression model is as follows: ∆*ITI (n*) = β_1_ * *ITI*_*Async *_ + β_2_ * ∆*ITI (n* *−* 1) + β_3_ * *ITI*_*Async*_ * ∆*ITI (n* *−* 1).
