# Supplementary material for: Paired Synchronous Rhythmic Finger Tapping without an External Timing Cue Shows Greater Speed Increases Relative to Those for Solo Tapping
Source: Sci Rep. 2017 Mar 9;7:43987. doi: 10.1038/srep43987 (PMC5343470; doi:10.1038/srep43987)
Supplement: Supplementary Materials [file srep43987-s1.pdf]

Supplementary materials for:

Paired Synchronous Rhythmic Finger Tapping without an External Timing Cue Shows Greater Speed Increases Relative to Those for Solo Tapping

Masahiro Okano, Masahiro Shinya, and Kazutoshi Kudo.

This PDF file includes:

Supplementary Analyses

Supplementary Figures

References

Supplementary Analysis

Effect of number of trials on ITI drift

To verify that data of trials within each tempo condition can be combined, we first conducted two-way repeated measures analysis of variance (ANOVA). We compared means for the final 30 ITI separately for solo and paired trials. Tempo (75, 120, and 200 bpm) and number of trials (1–3 for solo and 1–2 for paired trials) were used as factors. The ANOVA revealed no significant main effect of the number of trials, (solo condition, $F(1.48, 34.10) = 2.87$, $p = .084$; paired condition, $F(1, 23) = 1.24$, $p = .277$). These results suggest that both in solo and paired conditions performances of participants were consistent across trials (Figure S1). Therefore, in the analyses presented in the main text, we compared mean values across trials within tempo and solo/paired conditions.

Asynchrony of tap timing between partners

If emergent leadership of faster participants led to accelerated tapping, the leader’s tap should consistently precede his or her partner’s tap. Timing asynchrony (the difference between tap timing of partners, not ITI_{Async} in the regression analyses) fluctuated around 0 ms (Figure S3). Figures S4-9 depict histograms of timing asynchrony for faster participants. The histograms

show no consistent bias toward negative values. Taken together, these results suggest that there were no significant effects of emergent leadership.

Effect of participant type on intra- and interpersonal timing modulation

To investigate whether participant type (slow or fast) affects intra- and interpersonal timing modulation, we compared coefficients of determination (R^2). R^2 values were obtained via single regression analyses of groups of faster and slower participants (Figure S11). For any tempo and regression model (A: $\Delta ITI(n) = \beta * \Delta ITI(n)$ and B: $\Delta ITI(n) = \beta * ITI_{Async}(n–1)$), no significant effect of the groups was observed (A: $t(23) = 1.29$, 0.41, and 0.30; B: $t(23) = 0.64$, 0.90, and 0.58 for 75, 120, 200 bpm respectively; $ps > .05$).

Supplementary Figures

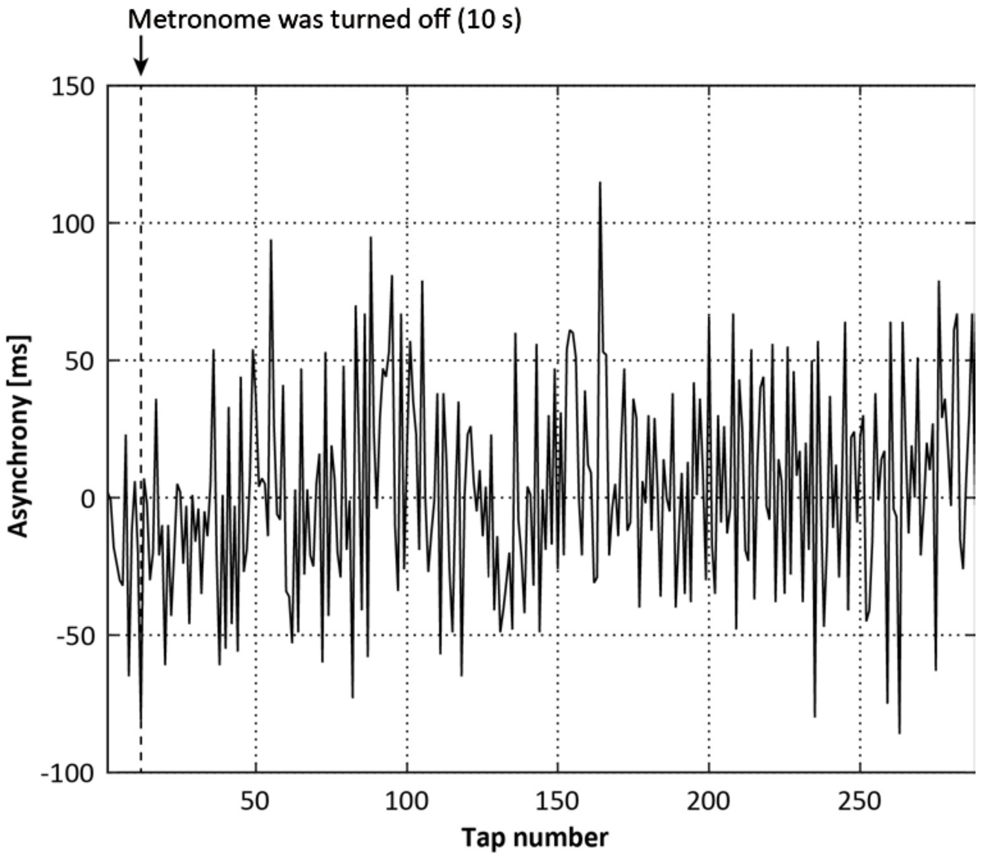

Figure S1. A typical example of an asynchrony time series for the faster participant. The Y-axis shows the difference between partners' tap timing. Note that the asynchrony measure differs from ITI_{Async} in the regression analysis. This figure is based on the same trial as Figure 1 in the main text. Asynchrony values fluctuate around 0 ms. Consequently, the amount of taps that precede or succeed the partner's tap are similar for both partners. For this trial, mean asynchrony was 2.39 ± 36.3 ms (mean ± SD).

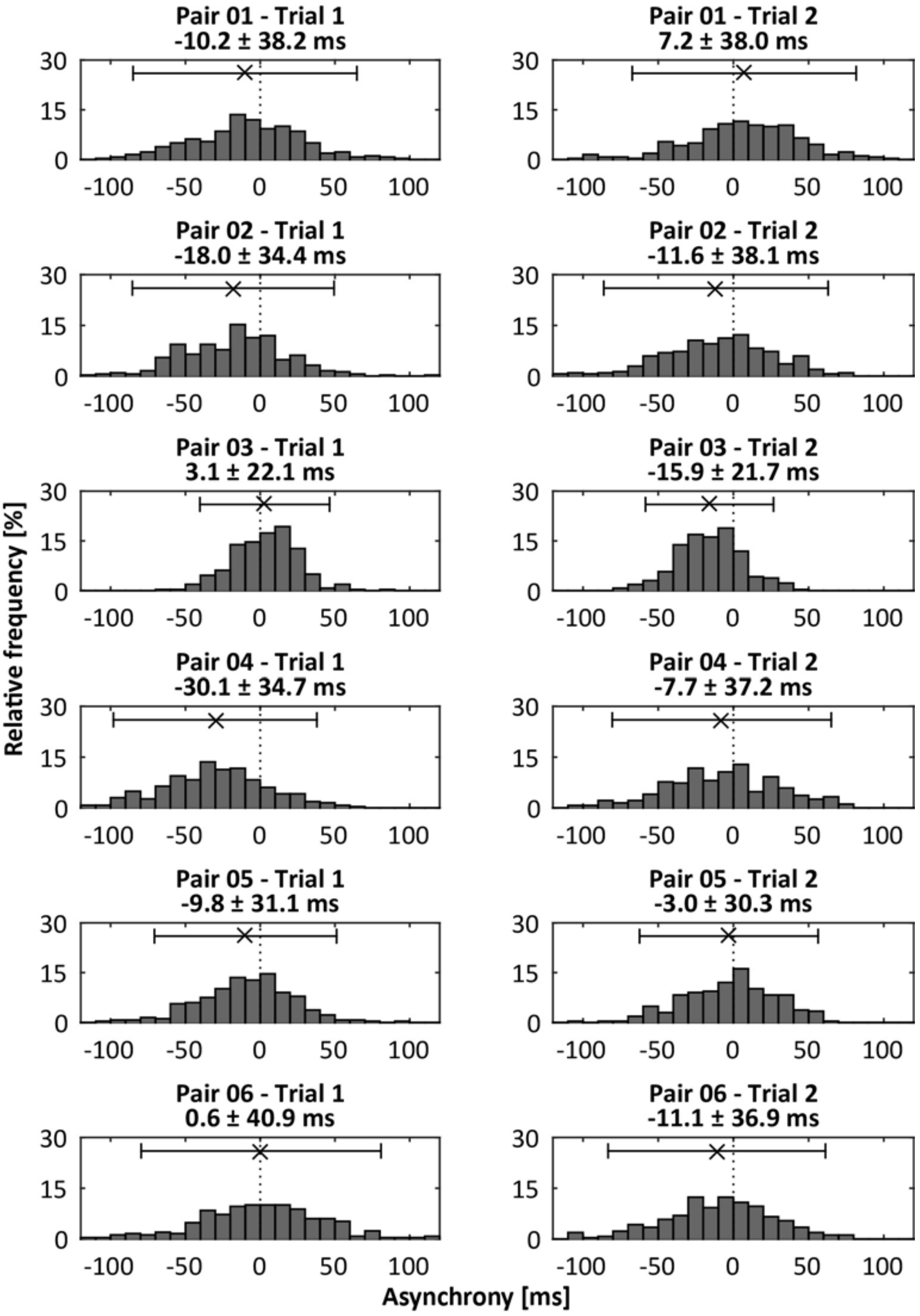

Figure S2. Histograms of asynchrony for faster participants in the 75-bpm paired conditions (pairs 1-6). Mean asynchrony values are indicated below pair and trial numbers above each histogram (mean ± SD). 95% prediction intervals are indicated above bars of each histogram.

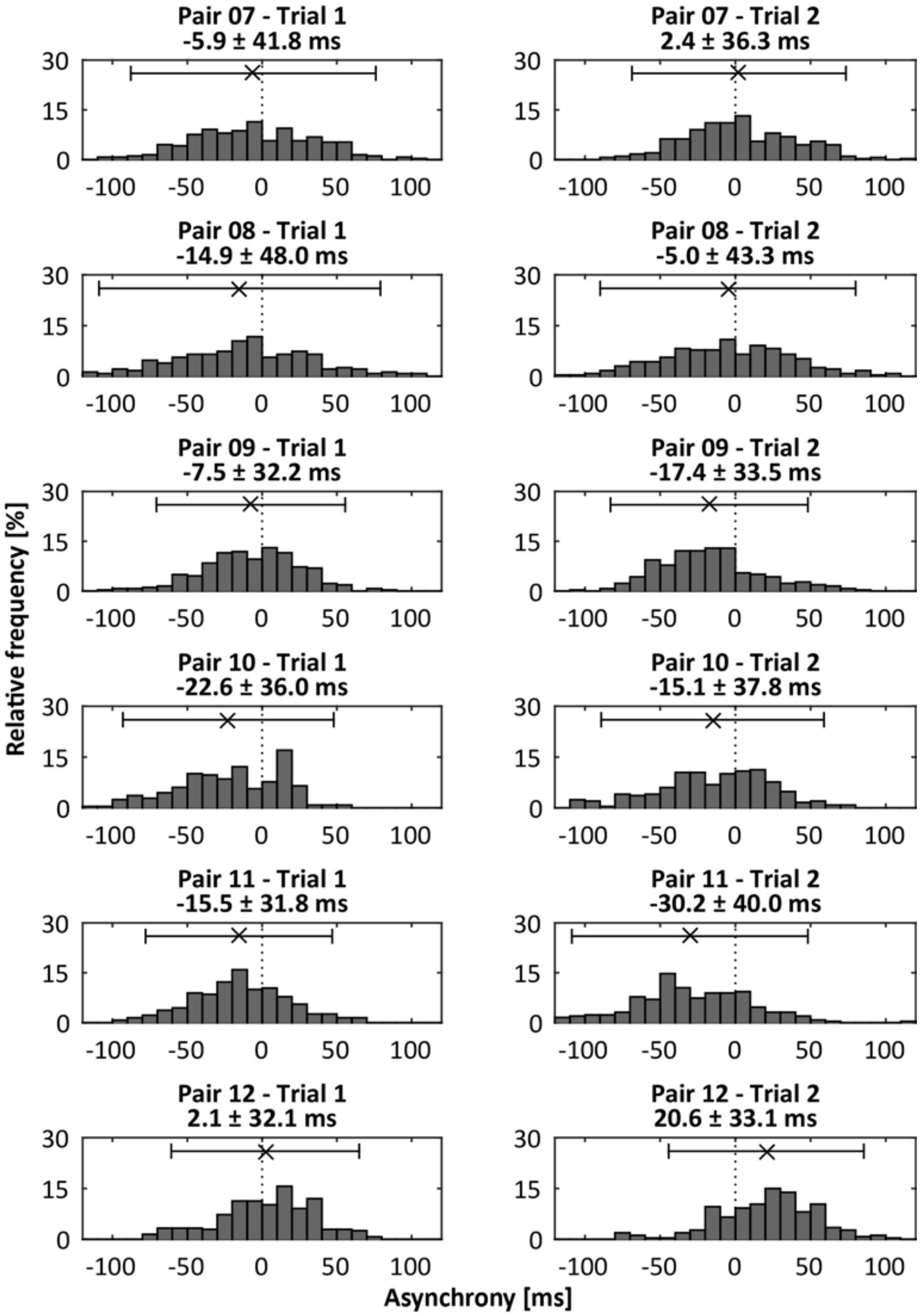

Figure S3. Histograms of asynchrony for faster participants in the 75-bpm paired conditions (pairs 7-12). Mean asynchrony values are indicated below pair and trial numbers above each histogram (mean ± SD). 95% prediction intervals are indicated above bars of each histogram.

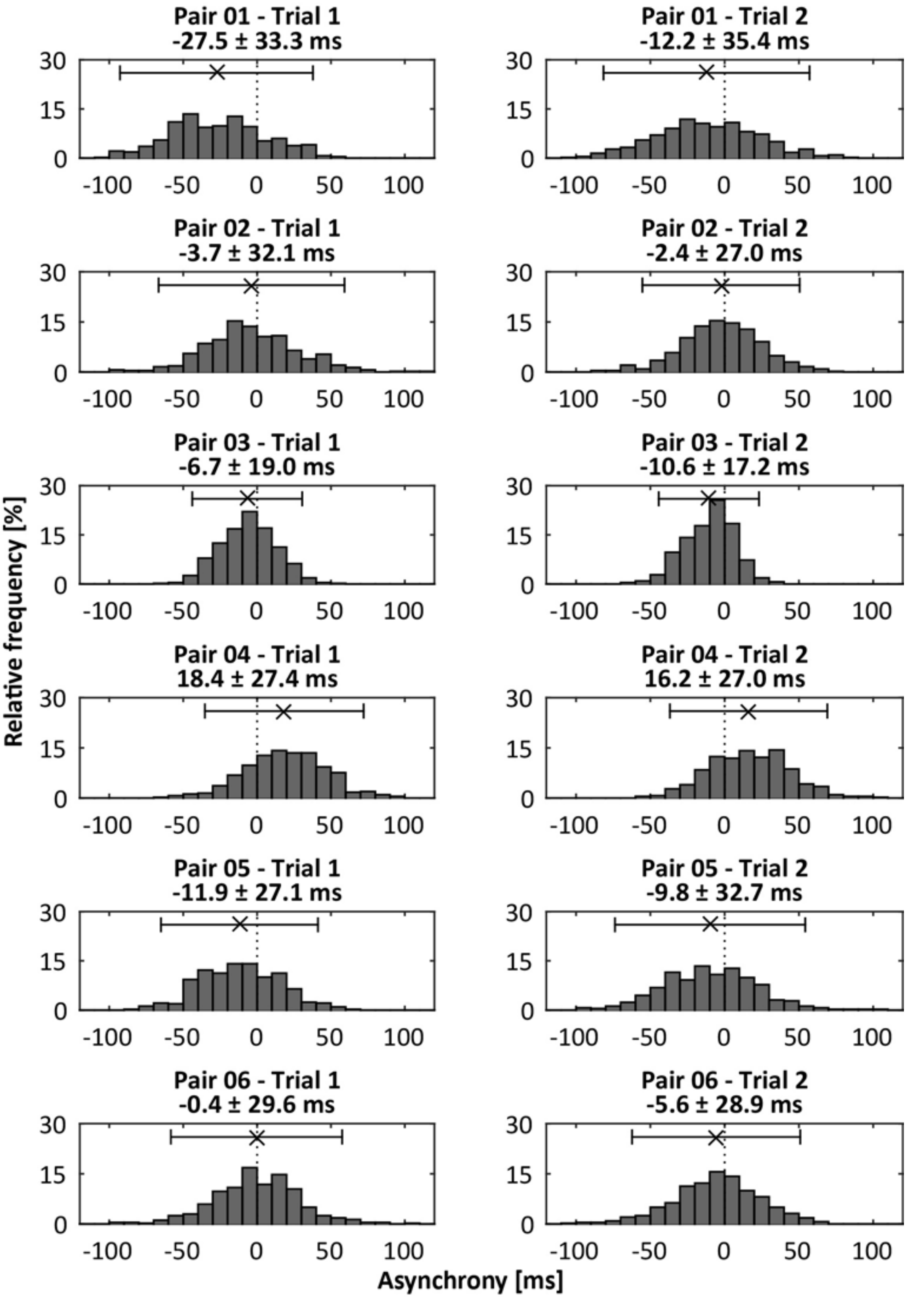

Figure S4. Histograms of asynchrony for faster participants in the 120-bpm paired conditions (pairs 1-6). Mean asynchrony values are indicated below pair and trial numbers above each histogram (mean ± SD). 95% prediction intervals are indicated above bars of each histogram.

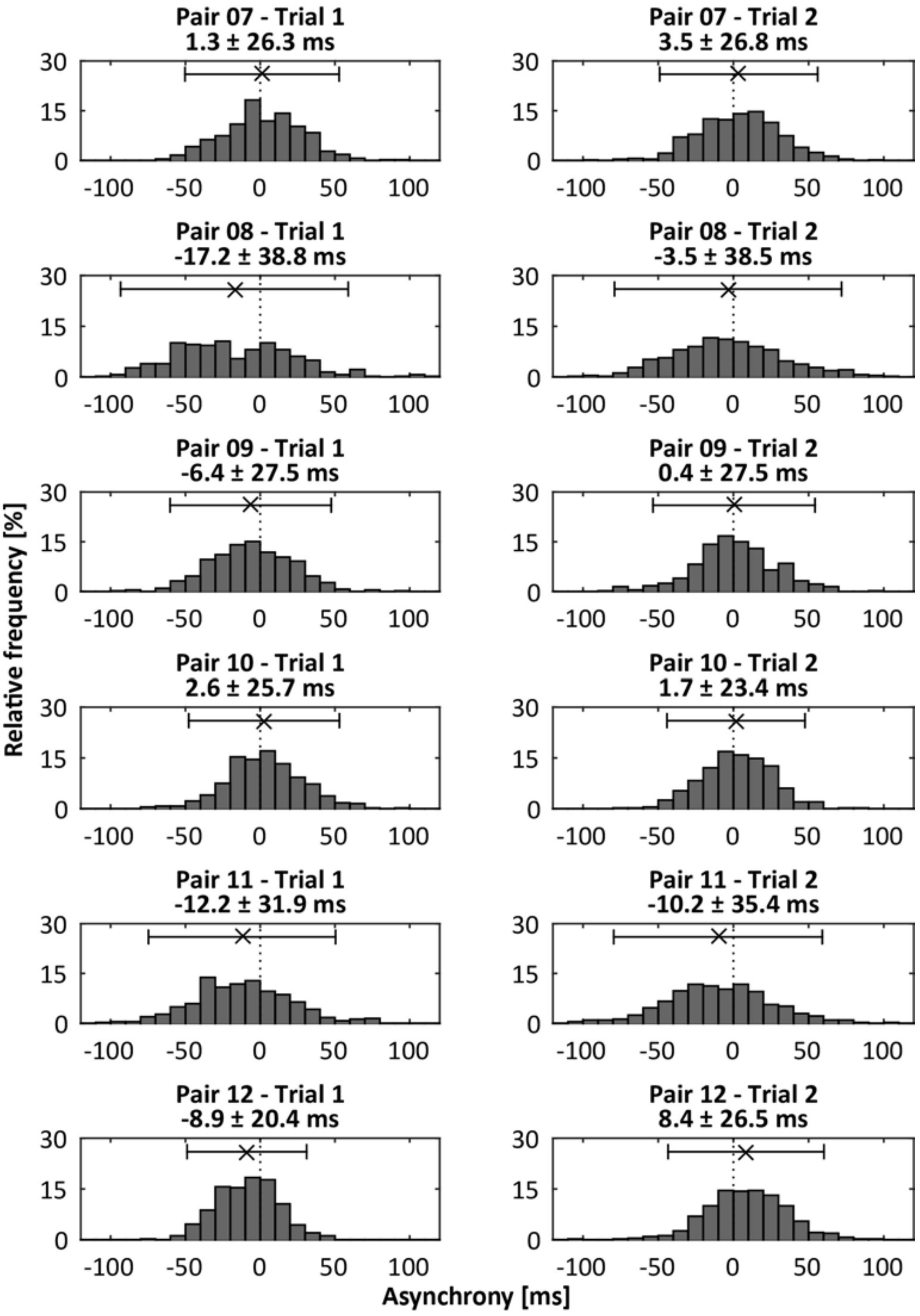

Figure S5. Histograms of asynchrony for faster participants in the 120-bpm paired conditions (pairs 7-12). Mean asynchrony values are indicated below pair and trial numbers above each histogram (mean ± SD). 95% prediction intervals are indicated above bars of each histogram.

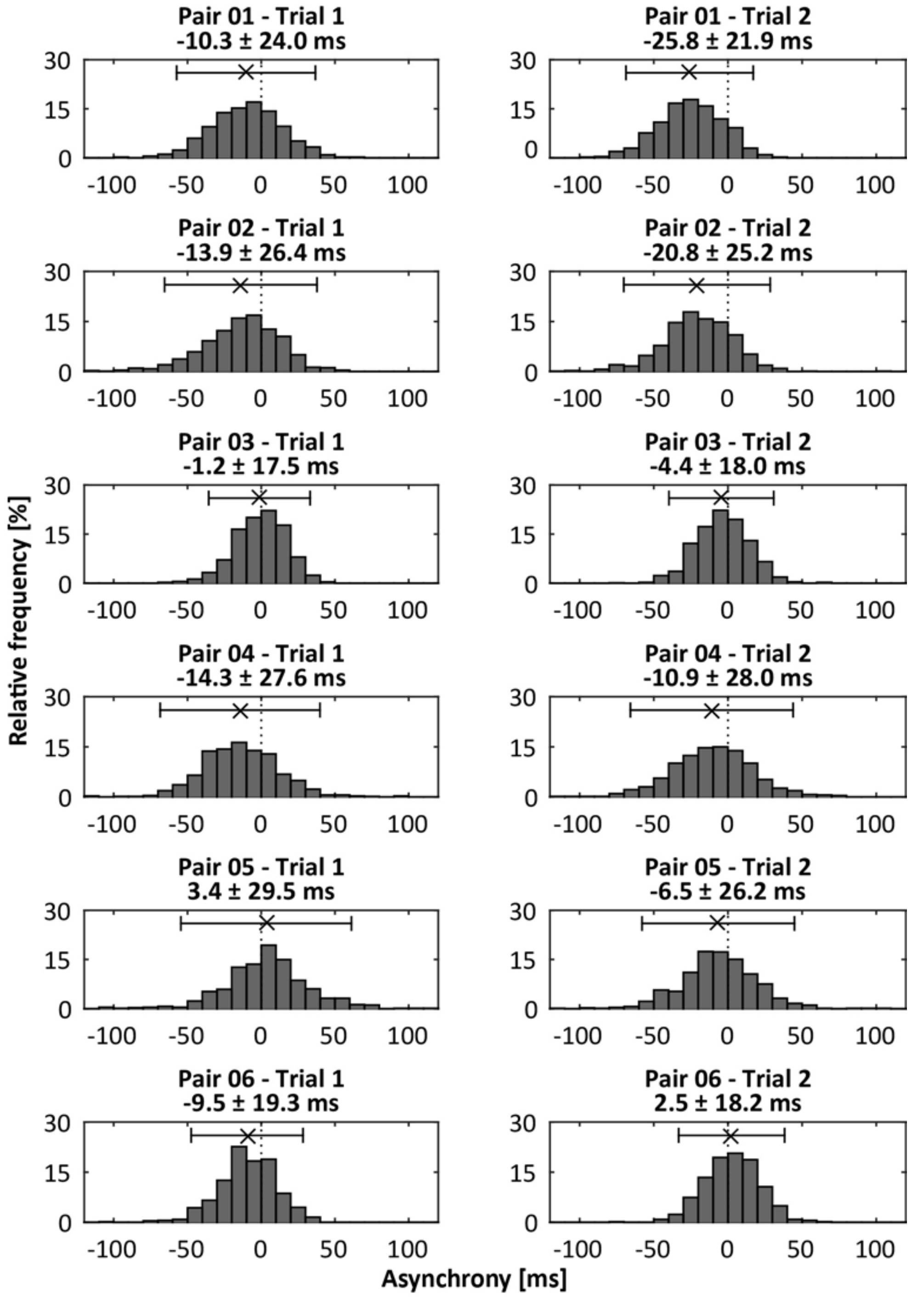

Figure S6. Histograms of asynchrony for faster participants in the 200-bpm paired conditions (pairs 1-6). Mean asynchrony values are indicated below pair and trial numbers above each histogram (mean ± SD). 95% prediction intervals are indicated above bars of each histogram.

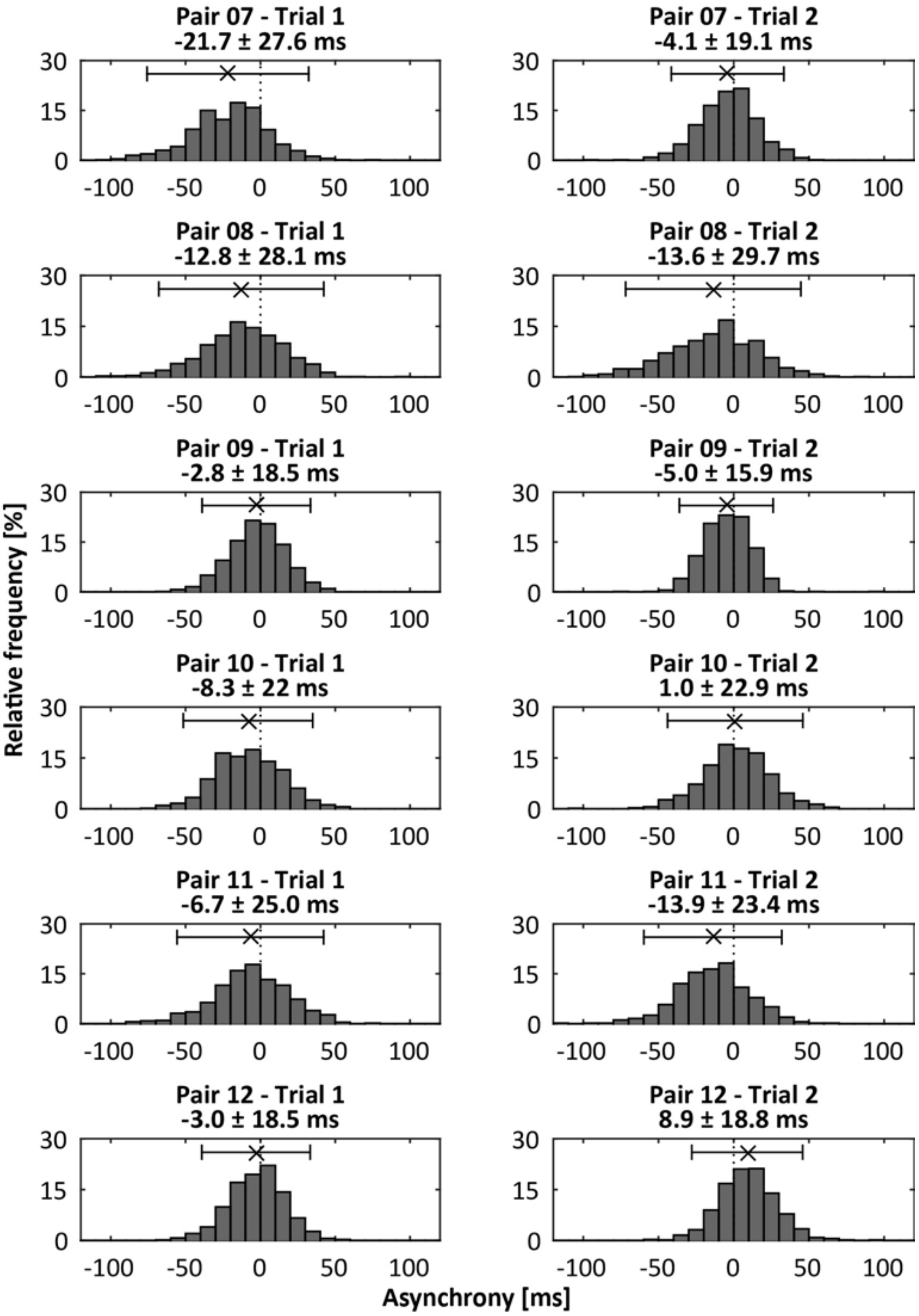

Figure S7. Histograms of asynchrony for faster participants in the 200-bpm paired conditions (pairs 7-12). Mean asynchrony values are indicated below pair and trial numbers above each histogram (mean ± SD). 95% prediction intervals are indicated above bars of each histogram.

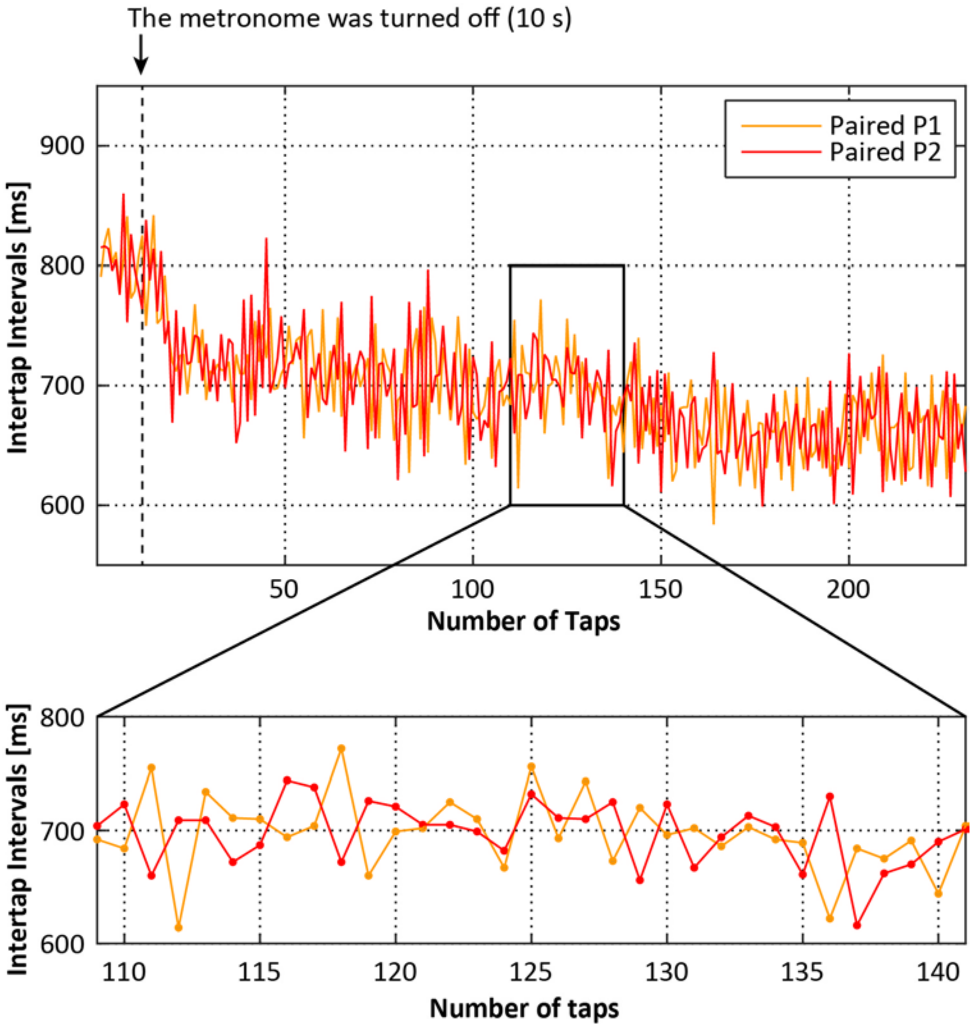

Figure S8. Hyper-follower pattern displayed as an ITI time series in paired conditions (same trial as Figure 1 in the main text). Konvalinka et al. reported negative lag-0 and positive lag±1 cross-correlation between ITI of partners in bidirectional coupling conditions[1]. The authors called partners "hyper-followers" i.e., partners followed each other's prior tap. Our data obtained from paired conditions showed a similar pattern.

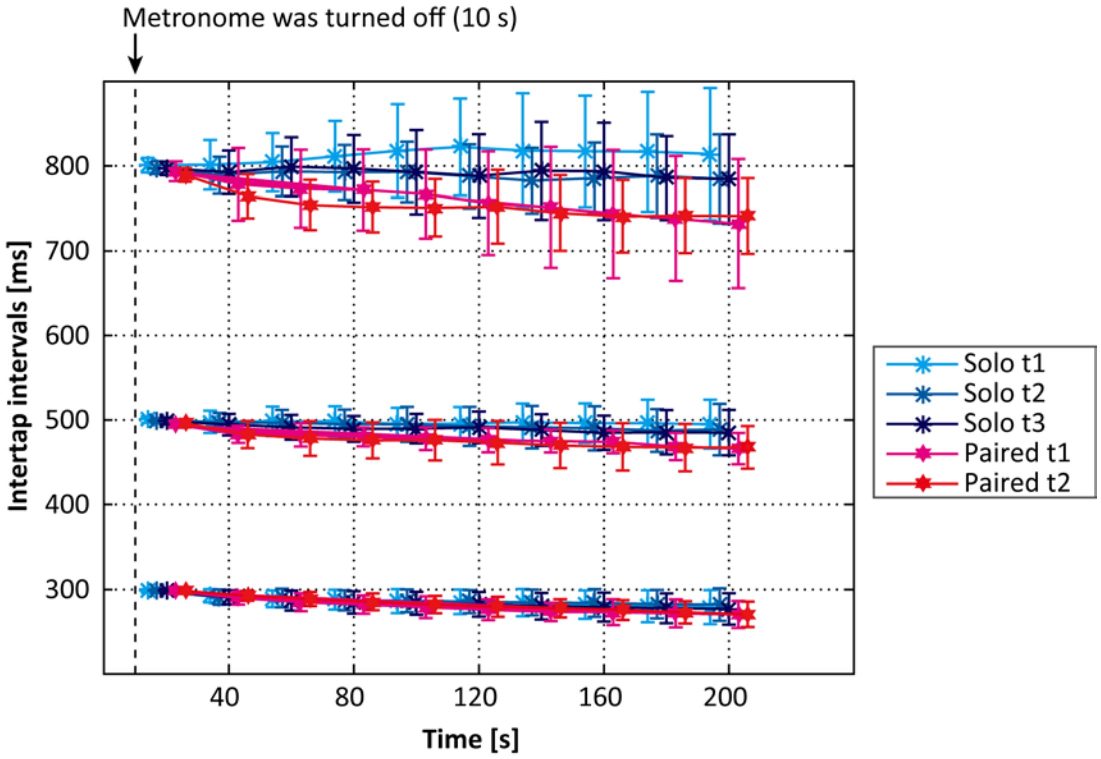

Figure S9. Mean ITI transition in 20-s intervals across all pairs for each trial. Mean ITI in solo trials are larger than in paired trials. The experiment started with a solo block. Then solo and paired blocks alternated. Therefore, ITI shortening in paired conditions relative to solo conditions cannot be explained by a change in performance due to factors such as fatigue.

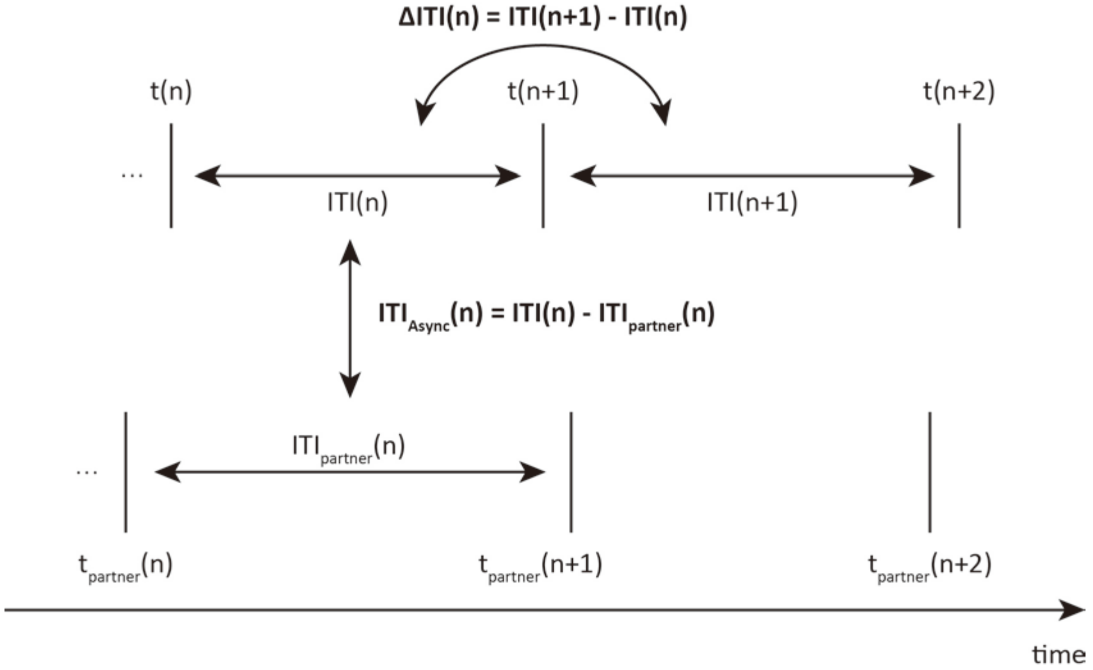

Figure S10. A diagram of the relationship between tap timing ($t(n)$, $\Delta ITI(n)$, and $ITI_{Async}(n\text{-}1)$).As denoted in equation (1)-(3), $ITI_{Async}(n)$ arises when the (n + 1)th tap occurred and ΔITI (n) arises when the (n + 2)th tap did; Consequently, the index for the ITI_{Async} immediately before the ΔITI (n) is n.

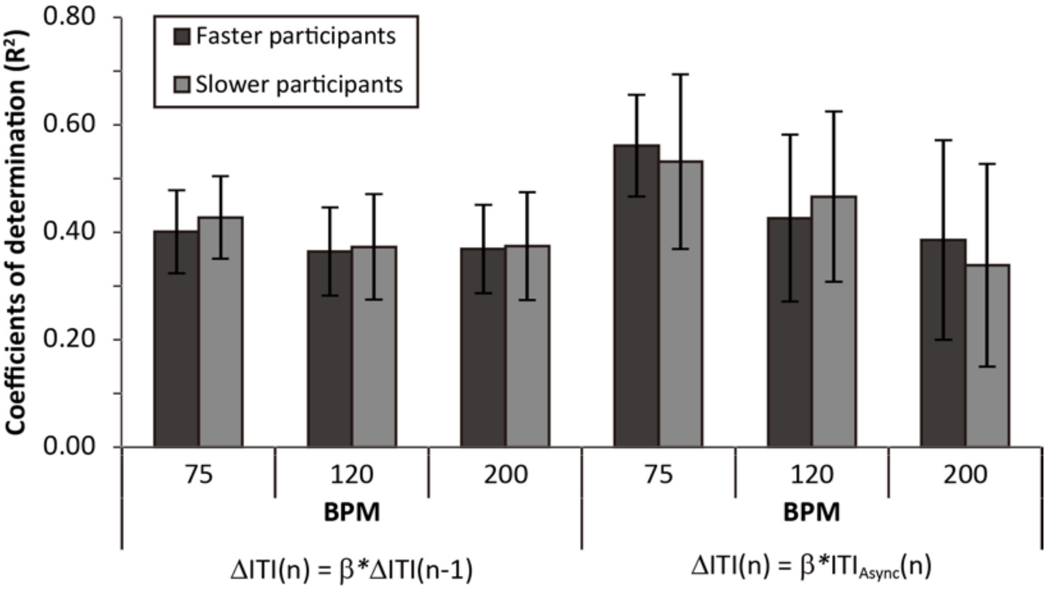

Figure S11. Coefficients of determination (R^2) for the paired conditions obtained via regression analyses for groups of faster and slower participants. There was no significant difference on R^2 between participant types (faster and slower participants).

References

1. Konvalinka, I., Vuust, P., Roepstorff, A. & Frith, C. D. Follow you, follow me: continuous mutual prediction and adaptation in joint tapping. *Q. J. Exp. Psychol. (Hove).* **63,** 2220–2230 (2010).
